# Association of hospitalization with structural brain alterations in patients with affective disorders over nine years

**DOI:** 10.1038/s41398-023-02452-z

**Published:** 2023-05-19

**Authors:** Katharina Förster, Dominik Grotegerd, Katharina Dohm, Hannah Lemke, Verena Enneking, Susanne Meinert, Ronny Redlich, Walter Heindel, Jochen Bauer, Harald Kugel, Thomas Suslow, Patricia Ohrmann, Angela Carballedo, Veronica O’Keane, Andrew Fagan, Kelly Doolin, Hazel McCarthy, Philipp Kanske, Thomas Frodl, Udo Dannlowski

**Affiliations:** 1 Institute for Translational Psychiatry, University of Münster, Münster, Germany; 2 Clinical Psychology and Behavioral Neuroscience, Faculty of Psychology, Technische Universität Dresden, Dresden, Germany; 3 Institute for Translational Neuroscience, University of Münster, Münster, Germany; 4 Department of Psychology, University of Halle, Halle, Germany; 5 Department of Radiology, University of Münster and University Hospital Münster, Münster, Germany; 6 Department of Psychosomatic Medicine and Psychotherapy, University of Leipzig Medical Center, Leipzig, Germany; 7 LWL-Hospital Muenster, Muenster, Germany; 8 Department of Psychiatry & Trinity College Institute of Neuroscience, University Dublin, Dublin, Ireland; 9 Department of Radiology, Mayo Clinic, Rochester, MN USA; 10 Department of Psychiatry, Psychotherapy and Psychosomatics, University Hospital Aachen, RWTH University Aachen, Aachen, Germany

**Keywords:** Depression, Neuroscience, Human behaviour, Prognostic markers

## Abstract

Repeated hospitalizations are a characteristic of severe disease courses in patients with affective disorders (PAD). To elucidate how a hospitalization during a nine-year follow-up in PAD affects brain structure, a longitudinal case-control study (mean [SD] follow-up period 8.98 [2.20] years) was conducted using structural neuroimaging. We investigated PAD (*N* = 38) and healthy controls (*N* = 37) at two sites (University of Münster, Germany, Trinity College Dublin, Ireland). PAD were divided into two groups based on the experience of in-patient psychiatric treatment during follow-up. Since the Dublin-patients were outpatients at baseline, the re-hospitalization analysis was limited to the Münster site (*N* = 52). Voxel-based morphometry was employed to examine hippocampus, insula, dorsolateral prefrontal cortex and whole-brain gray matter in two models: (1) group (patients/controls)×time (baseline/follow-up) interaction; (2) group (hospitalized patients/not-hospitalized patients/controls)×time interaction. Patients lost significantly more whole-brain gray matter volume of superior temporal gyrus and temporal pole compared to HC (*p*_FWE_ = 0.008). Patients hospitalized during follow-up lost significantly more insular volume than healthy controls (*p*_FWE_ = 0.025) and more volume in their hippocampus compared to not-hospitalized patients (*p*_FWE_ = 0.023), while patients without re-hospitalization did not differ from controls. These effects of hospitalization remained stable in a smaller sample excluding patients with bipolar disorder. PAD show gray matter volume decline in temporo-limbic regions over nine years. A hospitalization during follow-up comes with intensified gray matter volume decline in the insula and hippocampus. Since hospitalizations are a correlate of severity, this finding corroborates and extends the hypothesis that a severe course of disease has detrimental long-term effects on temporo-limbic brain structure in PAD.

## Introduction

A subgroup of patients with affective disorders (PAD) has a severe course of disease with repeated hospitalization: While approximately 20% of all patients with major depressive disorder (MDD) show a severe and recurrent course of disease, nearly 50% of patients with bipolar disorder (BD) show severe disease courses with recurrent episodes [1–4]. Longitudinal structural neuroimaging provides insights into the underlying neural mechanisms of severe courses of disease in PAD. In the long term, understanding these neural mechanisms could aid the prevention of severe disease courses and thus improve treatment of severe affective disorders.

Structural brain alterations in PAD have already been demonstrated by cross-sectional studies in frontal, temporal and limbic regions such as the dorsolateral prefrontal cortex (dlPFC), insula and hippocampus [5–13]. In particular, lower volume in the dlPFC and hippocampus has been linked to characteristics of the previous disease course such as the lifetime number of hospitalizations or the number of manic or depressive episodes [14–17].

However, only prospective studies allow to approximate causal conclusions about the relationship between structural brain changes and the course of disease. Unfortunately, only very few longitudinal studies focused on the interplay of gray matter changes and the course of disease [18–20]. These studies revealed greater brain volume loss in PAD compared to healthy control participants (HC), in particular in temporo-limbic areas such as the hippocampus and insula as well as parts of the frontal cortex (e.g., dlPFC) [21–24]. More recently, longitudinal studies have also established a link between these volume declines and recurrent manic [25] and depressive episodes [26].

In these few longitudinal studies, patients’ course of disease in concordance with gray matter alterations has only very rarely been investigated over time periods longer than 6 years [23, 27]. These studies also corroborate the idea that a detrimental disease course is associated with fronto-limbic volume decline. For instance, they have shown that the number of depressive relapses is associated with reduced insular volume [23]. However, most of the studies with longer follow-up intervals contained significant methodological limitations reducing their impact and generalizability, such as the use of different MRI scanners at baseline and follow-up [27], the investigation of an elderly sample [23], or the lack of a control group [25]. Still, conclusive neuroimaging studies with substantially longer follow-up intervals are needed to investigate how relevant features of a severe disease course affect gray matter volume.

Previous longitudinal structural MRI studies have mostly used the number of episodes as a correlate of severe disease courses. Number of episodes are usually based on retrospective patient reports. However, retrospective patient reports of depressed episodes may have questionable reliability and validity [28] specifically over longer time periods. For such longer follow-up periods, objective markers of a severe disease courses are needed. The number and duration of hospitalizations provide such criteria, since they are verifiable by medical reports.

The aim of this article is to elucidate the relationship between brain structural alterations and particularly severe disease courses in PAD in both frontal and temporo-limbic areas as well as on a whole-brain level. For this purpose, at least one hospitalization during follow-up was considered as a characteristic of a severe course of disease. To elucidate how these characteristics are associated with changes in brain structure, brain structural alterations were observed over nine years with a focus on the hippocampus, insula and dlPFC.

To tackle these research gaps, PAD were investigated at two study sites (Münster, Dublin). We hypothesize that (a) patients lose more volume over time than controls in the insula, hippocampus and dlPFC and (b) patients with a hospitalization until follow-up will lose more volume than HC and patients without re-hospitalization in these regions.

To allow for specific conclusions regarding the impact of hospitalization as a specific correlate of severe disease courses within affective disorders, we also explore the impact of important other correlates of severe disease courses, for instance, comorbid mental disorders, the number of depressive episodes during the study interval, the intake of psychotropic medication, baseline depressive symptom-severity, baseline number of hospitalizations on our effects, as well as the inclusion of patients with BD.

## Materials and methods

### Participants

Eighty-seven participants took part in our longitudinal structural MRI investigation across sites (Münster *N* = 61, Dublin *N* = 26). All participants gave written informed consent according to the Declaration of Helsinki. Inclusion criterion for PAD was a lifetime diagnosis of BD or MDD at follow-up. Patients with dysthymia were not included in the analysis. Next to the general exclusion criteria for MRI studies such as metal implants, we also excluded participants with severe cardiovascular diseases, unstable diabetes, life-time alcohol-dependence and substance dependence at follow-up, as well as severe neurological diseases such as Parkinson’s disease, stroke, and dementia. After quality control of the imaging data, *N* = 52 participants (28 PAD: BD = 6, MDD = 22; HC = 24) remained in the Münster sample as well as *N* = 23 participants (10 PAD: MDD = 10; HC = 13) in the Dublin sample (see Table 1 and Appendix A: Supplementary Table 1, Supplementary Table 2, Supplementary Table 3). The focus of this analysis were the effects of diagnosis and course of affective disorder on brain volume loss. Because of the unique follow-up length and to increase sensitivity for this analysis, patients with both, BD and MDD, were included. To also increase specificity of conclusions regarding MDD-patients, the analysis was repeated excluding patients with BD (total *N* = 69, *N* = 32 MDD-patients, *N* = 37 HC).

**Table 1:** Sample characteristics of the Münster-dublin longitudinal cohort.

	Healthy controls (*N* = 37) mean (SD)		MDD patients (*N* = 32) mean (SD)		BD patients (*N* = 6) mean (*SD*)		Group ANOVA	
	Baseline	Follow-up	Baseline	Follow-up	Baseline	Follow-up	*p* value	Post hoc
Age	33.73 (12.97)	42.65 (12.63)	37.66 (10.62)	46.23 (9.87)	34.00 (7.51)	44.33 (7.06)	.386	-
FU-interval (in months)	/	107.89 (28.55)	/	104.09 (24.13)	/	126.50 (28.31)	.163	-
Clinical characteristics								
BDI-I	2.49 (2.29)	2.40 (2.59)	23.50 (11.67)	10.50 (8.86)	32.40 (5.68)	12.50 (8.36)	<.001	HC < MDD HC < BD
HAM-D	/	1.71 (3.20)	/	6.34 (7.03)	/	6.00 (8.88)	.004	HC < MDD HC < BD
YMRS	/	0.57 (1.04)	/	0.77 (0.92)	/	0 (0.00)	.205	-
Number of hospitalizations	/	/	1.61 (1.46)	0.91 (1.51)	3.50 (1.98)	4.33 (6.95)	.004	BD > MDD
Duration of hospitalizations (in weeks)	/	/	4.65 (4.11)	2.18 (4.12)	15.08 (11.33)	11.00 (13.91)	.001	BD > MDD

Hospitalization data, data on patients with bipolar disorder and young mania rating scale are only available for the Münster site. Hamilton depression rating scale and young mania rating scale at baseline were unavailable for a majority of participants and are therefore not reported. *P* values are reported for ANOVA with subsequent post hoc *t* tests and between-subjects contrasts of a repeated measurements ANOVA if measurements at baseline and follow-up were available.

*HC* healthy controls, *MDD* major depressive disorder, *BD* bipolar disorder, *FU-interval* follow-up interval *BDI-I* Beck depression inventory; *HAM-D* Hamilton depression rating scale, *YMRS* Young Mania Rating Scale.

In Münster, all patients were recruited during in-patient treatment at the Department of Psychiatry at the University of Münster at baseline (BD = 8, MDD = 27, HC = 26). Diagnoses were made by a clinical psychologist or psychiatrist at baseline and reviewed by a second clinician at baseline and follow-up using the SCID-I interview according to DSM-IV criteria [29]. HC were recruited from the local community and by newspaper announcements in Münster. Inclusion criterion was the absence of any lifetime mental disorder at baseline or follow-up. A SCID-I interview verified the absence of lifetime diagnosis. All participants have been taking part in cross-sectional studies conducted by our lab between 2005–2008 and are part of the Münster Neuroimaging Cohort [30–34]. During follow-up, one MDD patient reported a hypomanic episode that led to a change in diagnosis from MDD to BD at follow-up. Two participants, initially healthy controls, were diagnosed with at least one depressive episode during the interval, leading to a change in diagnosis from HC to MDD. For the analysis participants were assigned to the group (patients vs. controls) using the diagnostic information of the follow-up. Descriptive data of the Münster sample can be found in Supplementary Table 2 (Appendix A).

In Dublin, 26 participants (MDD = 12, HC = 14) were investigated at follow-up, approximately six years after baseline measurements. At baseline, all patients were undergoing outpatient treatment at the mental health services of Tallaght Hospital, Dublin and St. James’s Hospital, Dublin, Ireland. MDD was clinically diagnosed by consultant psychiatrists based on DSM-IV criteria using the SCID-I. HCs were recruited from the local community in Dublin. Descriptive data of the Dublin sample can be found in Supplementary Table 3 (Appendix A).

### Measures

Self-rated and observer-rated scales were completed for all participants at both times points. These included the Hamilton Depression Rating Scale (HAM-D) [35] and Beck’s Depression Inventory (Münster: BDI-I, Dublin: BDI-II) [36] At follow-up, full remission was defined according to DSM-IV criteria (two months without depressive symptoms) in combination with a HAM-D Score lower than 8 (see also Table 1).

Severity of the course of disease was determined by at least one hospitalization within the follow-up interval. Hospitalization during follow-up was determined by a list of patients’ lifetime hospitalizations with date, duration, diagnosis, and hospital based on their medical reports. Patients reported exact dates based on their medical reports or brought their medical reports for verification. Hence, hospitalization were objective and verifiable characteristics of a severe disease course. Reason for hospitalization was treatment of a mood episode during BD or MDD. PAD that had at least one psychiatric in-patient treatment during follow-up are referred to as “patients with hospitalization”, while those without in-patient treatment during follow-up were considered as “patients without hospitalization”. Since the Dublin patients were outpatients, the analysis regarding the effects of hospitalization in the interval was limited to the Münster sample (*N* = 52).

We collected a list of current medication intake at baseline and follow-up. Psychotropic medication was measured at follow-up using a medication load index [37]: Each psychotropic medication was coded as absent = 0, low = 1 (equal or lower average dose), or high = 2 (greater than average dose), relative to the midpoint of the daily dose range recommended by Physician’s-Desk-Reference [38]. To build the index, all medication codes per participant and time point were summed, which finally yielded a composite measure of total medication exposure for each subject at follow-up (medication load index).

Details of MRI data collection and preprocessing can be found in the supplements (Appendix B).

### Statistical analysis

Longitudinal VBM analyses were conducted on gray matter segments in SPM12 using an absolute threshold masking of 0.1. We used a flexible-factorial design and conducted three 2×2/3 × 2 analyses of covariance (ANCOVA) with group as between-participant factor and time (baseline and follow-up) as within-participant factor controlling for age. In *model 1*, we investigated the effect of diagnosis on gray matter volume with a directed group (AD-patients, HC) × time contrast: We hypothesized that PAD lost more volume during the follow-up than HC. In *model 2*, limited to the Muenster sample only, we investigated another directed group (patients with hospitalization, patients without hospitalization, HC) × time interaction on gray matter volume to determine the effects of severe disease courses on gray matter volume loss over time according to our a priori hypothesis. In another supplemental model (*model 3*), we used a different definition of severe disease course and compared the brain structural development of remitted and unremitted patients at follow-up to HC (see Appendix C). Main effects of group were estimated using post hoc tests controlling for age, sex and site (see Appendix D).

We conducted three region of interest (ROI)-analysis on the bilateral hippocampus, insula and dlPFC, as well as an exploratory whole-brain analysis. ROIs were determined using the AAL-atlas implemented in the Wake Forest University Pickatlas (http://fmri.wfubmc.edu/software/PickAtlas). Anatomical labeling of significant VBM clusters in the whole-brain analysis was performed by the AAL toolbox (release 3v1 [39, 40]). We used a rigorous statistical threshold of *p* < 0.05 (one-tailed), familywise error (FWE) corrected on the voxel level. Exploratory results are reported at a statistical threshold of *p* < 0.001, uncorrected, with a cluster threshold of *k* = 300 voxels (see Appendix E, Supplementary Table S4).

We performed spearman correlation analyses to investigate potentially confounding effects of psychotropic medication using the medication load index at follow-up as well as baseline symptom severity and the number of hospitalizations before baseline, the number of episodes as well as lifetime comorbid mental disorders on gray matter volume differences in the patient subsamples (see Appendix F, Supplementary Table S5). Therefore, VBM gray matter volume differences were computed by subtracting contrast values extracted at baseline from those at follow-up.

## Results

### Model 1: volume decline in patients compared to healthy participants

Model 1 included 38 PAD (20 women and 18 men, mean [SD] age, 37.08 [10.19] years) and 37 HC (25 women and 12 men, mean [SD] age, 33.97 [12.71] years, see also Table 1, Supplementary Table 1 and Supplementary Table 2 in Appendix A). Main effects of time and group are reported in Appendix D.

The ROI analysis on hippocampal volume revealed a significant group × time interaction: Patients lost significantly more volume than controls bilaterally in the hippocampus (left: t(71) = 4.48, *p*_FWE_ = 0.001, *k* = 183, *x* = −21, *y* = −9, *z* = −12; right: *t*(71) = 4.28, *p*_FWE_ = 0.008, k = 57, *x* = 16, *y* = −4, *z* = −16). The ROI analysis on insular volume revealed a significant group × time interaction. Patients lost significantly more volume than controls in three clusters of the right insula (*t*(71) = 4.68, *p*_FWE_ = 0.004, *k* = 85, *x* = 42, *y* = −15, *z* = −2; *t*(71) = 4.34, *p*_FWE_ = 0.004; *k* = 54, *x* = 27, *y* = 12, *z* = −20; *t*(71) = 4.1, *p*_FWE_ = 0.018, k = 33, *x* = 51, *y* = 2, *z* = −2).

The ROI analysis on the dlPFC revealed no significant results (*p*_FWE_ = 0.552).

**Fig. 1:**
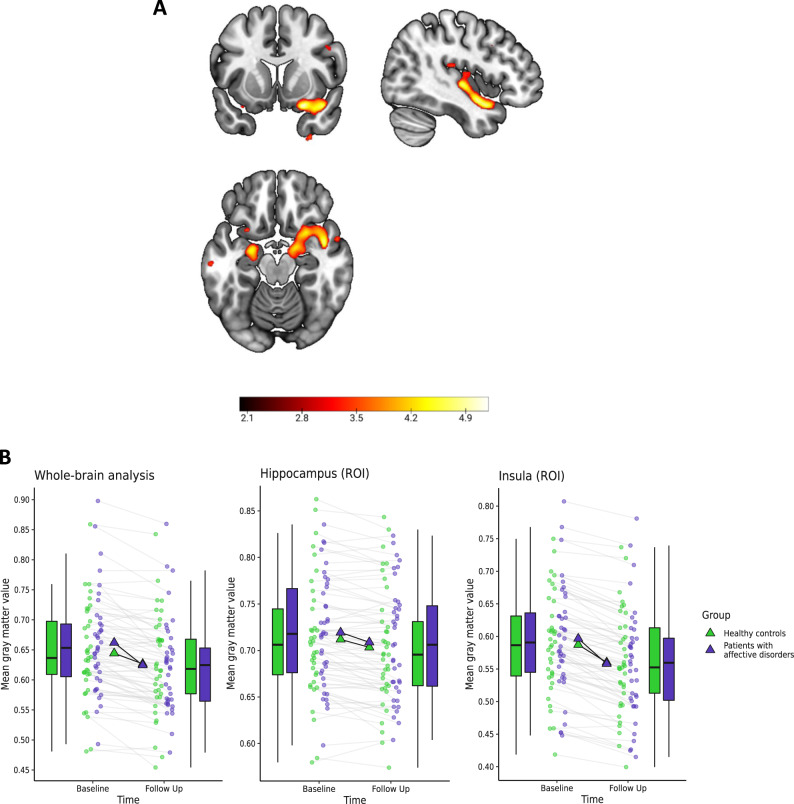
Significant group x time interaction in the diagnosis model (model 1). **A** Depicted is the whole-brain cluster of the groupxtime interaction, for display reasons, the statistical threshold was set to *p* < 0.001, uncorrected, color bar indicates *t*-values of the group by time contrast of the comparison patients vs. controls (model 1), extracted at the peak voxel: *x* = 24, *y* = 9, *z* = −21. **B** Contrast values were extracted at the peak voxel (hippocampus: *x* = −21, *y* = −9, *z* = −12, insula: *x* = 42, *y* = −15, *z* = −2, whole-brain analysis: *x* = 24, *y* = 9, *z* = −21) and depicted for patients and healthy participants: patients show a steeper reduction of gray matter volume of the whole-brain cluster and the regions of interest (ROI) hippocampus and insula. Graph shows mean gray matter values (mean and individual data points) extracted from the flexible-factorial model at baseline and follow-up with a boxplot (median, first and third quartile) to illustrate the distribution of the data.

The whole-brain analysis revealed a significant group × time interaction in the right temporal lobe, showing that patients lost more volume than HC (temporal pole: t(71) = 5.25, *p*_FWE_ = 0.018, *k* = 14, *x* = 24, *y* = 9, *z* = −21; superior temporal gyrus: t(71) = 5.05, *p*_FWE_ = 0.042, *k* = 3, *x* = 44, *y* = −2, z = 15; see Fig. 1). An exploratory analysis (*p* < 0.001, *k* = 300) revealed that this cluster extended into the right insula and hippocampus, further corroborating our results from the ROI analysis on a whole-brain level. The results of this exploratory whole-brain analysis can be found in the supplements (Appendix E).

### Model 2: effects of hospitalization as a correlate of severe disease course

Model 2 contained the Münster sample: 13 patients with hospitalization during follow-up (7 women and 6 men; mean [SD] age, 47.31 [10.98] years) and 15 patients without hospitalization (6 women and 9 men-, mean [SD] age, 42.47 [8.68] years) as well as 24 HC (16 women and 8 men; mean [SD] age, 40.21 [10.47] years, see Supplementary Table 1 in Appendix A).

**Fig. 2:**
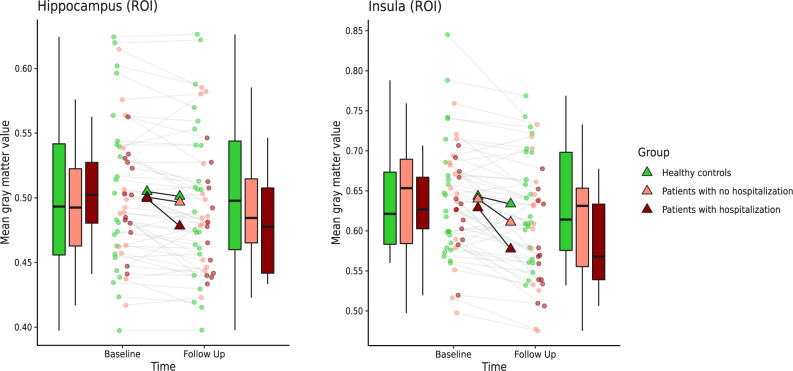
Significant group x time interaction in the hospitalization model (model 2). Differences in brain volume decline between the patient groups according to their course of disease and healthy participants: We extracted the contrast values of the significant region of interest (ROI) analysis of the hippocampus and insula. Here, the hippocampus was yielding a significant group x time interaction for the patients with hospitalization in comparison to the patients without hospitalization and for the insula a significant group x time interaction for the patients with hospitalization in comparison to the controls. Both contrasts were extracted at the peak voxel (hippocampus: *x* = −23, *y* = −10, *z* = −21, insula: *x* = 34, *y* = 12, *z* = −16). Graph shows mean gray matter values (mean and individual data points) extracted from the flexible-factorial model at baseline and follow-up with a boxplot (median, first and third quartile) to illustrate the distribution of the data.

While patients with hospitalization lost more volume in the ROI analysis of the right insula (t(49) = 3.92, *p*_FWE_ = 0.025, *k* = 29, *x* = 34, y = 12, *z* = −16) in comparison to HC, patients without hospitalization did not differ significantly from HC (all *p*_FWE_ > 0.26) or hospitalized patients in their insula volume loss (all *p*_FWE_ > 0.32, see Fig. 2). The ROI analysis of the hippocampus indicated that patients with hospitalization lost more volume than HC and patients without hospitalization (*t*(49) = 4.03, *p*_FWE_ = 0.023, *k* = 25, *x* = −23, *y* = −10, z = −21). The ROI analysis of the dlPFC yielded no significant results (all *p*_FWE_ > 0.96). The whole-brain analysis revealed no significant results (all *p*_FWE_ > 0.53).

### Effects of confounders

Gray matter changes significantly correlated with the number of depressive episodes in the interval. This is an effect that can be expected since both high recurrence and repeated hospitalization are both correlates of a severe disease course. Gray matter changes were not significantly correlated with previous course of disease or initial symptom severity at baseline. Gray matter changes were also not correlated with the intake of psychotropic medication or did not differ between patients with BD and MDD in both models (see Appendix F, Supplementary Table S5), as well as patients reporting comorbidities or not. To also test the specificity of our results to patients with depression, we excluded patients with BD in a secondary analysis (see Appendix G).

## Discussion

This study investigated the association of hospitalization as a feature of severe disease courses of PAD with brain structural changes over nine years. In our study, PAD lost more volume in a cluster in the temporal lobe extending into the hippocampus and insula in comparison to HC. Most importantly, patients with hospitalization until follow-up lost more volume in the insula and hippocampus in comparison to HC. In contrast, patients without further hospitalization did not differ from HC in their brain volume decline of insula and hippocampus. Thus, we were able to confirm and extend our earlier findings that volume decline merely appeared in patients with a worse disease outcome [21, 26]. We carefully controlled our findings for associations with putative confounders: Since there was no correlation between the number of hospitalizations that happened before baseline measurements and volume decline between baseline and follow-up, we could rule out that the brain structural alterations result from the disease course before the patients took part in our study. The effects of hospitalization on brain structure were also replicated after excluding BD-patients from the analysis (see Appendix G). While MDD-patients and HC did not differ in their brain structural changes, hospitalized MDD-patients lost significantly more hippocampal and insular volume to follow-up than HC. Moreover, MDD-patients with hospitalization also lost more hippocampal volume than MDD-patients without hospitalization during follow-up, corroborating the stability of our findings in MDD-patients. We also replicated our results by using remission at follow-up to group patients in the entire sample (*n* = 75) and showed that the number of episodes in the interval was correlated with insular volume loss as another marker for a severe disease course. These additional analyses indicate that brain volume loss is linked to a severe course of affective disorders independent of the correlate that is used (follow-up remission, re-hospitalization or number of depressive episodes).

While all participants lost volume over time, surprisingly, PAD did not show lower gray matter volume than healthy controls across time points. This fits well with findings indicating that differences in gray matter volume between patients with depression and HC are rather small [12, 41] and that extremely large neuroimaging samples are needed to detect these subtle gray matter volume differences in cross-sectional analyses. At this point, our results further highlight the importance of longitudinal studies in structural neuroimaging of affective disorders. Our results show that cross-sectional quantitative differences in gray matter volume cannot be demonstrated between patients and controls in our sample, but that development over time between both groups can very well be quantitatively delineated. Moreover, our results associate progressive gray matter decline with hospitalization as a feature of a severe disease course rather than with the diagnosis itself.

The observed gray matter loss in temporo-limbic areas extend previous cross-sectional observations indicating a negative association between the lifetime number and duration of hospitalization, the lifetime number and duration of depressive episodes and hippocampal volume [17, 42] as well as the association between relapse and volume decreases of the insula, hippocampus and temporal lobe [21, 23, 26].

We investigated gray matter volume loss using voxel-based morphometry, which among other techniques enables us to detect gray matter changes of the brain that could result from changes in neural organization (e.g., glial cell density, neuron size or dendritic branching) [43–45]. These changes may in turn affect the functional neuronal circuitry of regions showing volume loss. Although we cannot form any firm conclusions regarding the functional role of this gray matter decline, gray matter loss in the hippocampus and insula might predispose or result from mood-congruent biased emotion processing in PAD and may intensify suppressive emotion regulation. Further speculating on the functional nature of this volume decline, it has been revealed that more and longer hospitalizations are also associated with lower activity in the insula and hippocampus during facial emotion processing [46], suggesting a decreased metabolic activity in emotion-processing areas as a result of severe disease course. Volume loss may predispose or result from a reduced propensity to process and regulate emotional experiences ultimately reinforcing disturbed affective states, that is depression or mania. This is also reflected by functional neuroimaging studies in PAD indicating a reduced capacity to inhibit and regulate limbic activity resulting in a hyperreactivity to mood-congruent stimuli during depression and (hypo-)mania [47–53].

Next to only little evidence on the impact of adverse disease courses characterized by multiple hospitalizations on brain structure over time, neurobiological processes in the brain during recovery from affective disorder remain unclear. Our results point towards the idea that a course of affective disorders without recurrent hospitalization may ultimately result in a normalized brain structural development, specifically in emotion-processing regions such as the insula and hippocampus. Here, future studies should focus on defining and monitoring recovery in PAD also on a brain structural level and elucidate if recovery is associated with brain structural normalization.

To the best of our knowledge, this is the first study investigating the effects of hospitalization as a feature of severe disease course on brain volume with a follow-up interval of almost a decade and thus the first study allowing for conclusions regarding the long-term effects of severity in affective disorders on brain structure.

### Limitations

Data on patients with BD were only available for the Münster site. To date, there is no study comparing structural brain alterations over time in patients with BD and MDD. Hence, we do not know whether the effects of severe course of disease on brain structure are a transdiagnostic phenomenon.

Unlike previous studies [17, 23, 46], the report of depressive and manic episodes in the interval was not used to measure the severity of disease course. Retrospectively reported mood episodes can be biased by the current mood state [28] and have a recall-rate lower than 50% over longer periods of time [54]. To increase reliability of these retrospective reports, electronic diaries of mood and life-charting have been suggested. Since patients with a very severe course of disease and significant impairment would probably not continue a mood diary for nine years, this approach would not have been feasible and suitable for the aim of our study. Since hospitalization data is accessible, verifiable and does not rely on patient effort and motivation, hospitalization during follow-up was used as a marker of severe disease course.

Given our recent findings [55] the sample size is too small for meaningful cross-sectional findings. However, our longitudinal within-subject analysis using a flexible-factorial model has an increased sensitivity compared to the cross-sectional analysis and thus allows viable conclusions. In comparison to previous longitudinal neuroimaging studies with a comparable follow-up length [23, 27] our sample size (*N* = 75) is the biggest so far.

## Conclusions

The present study revealed detrimental effects of hospitalization as a marker of a severe disease courses on brain structure in PAD using a longitudinal design. Observed volume decline in patients from baseline to follow-up affected brain structures involved in emotion processing and regulation. Our results highlight the association between hospitalization as a feature of long-term disease progression and morphologic brain alterations in PAD. This study demands a more fine-grained analysis of long-term disease progression and its interaction with brain structure.

## Supplementary information

Supplemental Material
