# Supplementary material for: Association of hospitalization with structural brain alterations in patients with affective disorders over nine years
Source: Transl Psychiatry. 2023 May 19;13:170. doi: 10.1038/s41398-023-02452-z (PMC10195797; doi:10.1038/s41398-023-02452-z)
Supplement: Supplementary file 1 — Supplemental Material [file 41398_2023_2452_MOESM1_ESM.docx]

**Supplemental Material**

**Appendix A.**

Sample characteristics for the hospitalization and the remission model (model 2 and 3)

Table S1. Metric sample characteristics of the patients samples in the remission and hospitalization model

Table S2. Sociodemographic, questionnaire and clinical data of study participants in Münster

Table S3. Sociodemographic, questionnaire and clinical data of study participants in Dublin

**Appendix B.**

Magnetic resonance imaging parameters

**Appendix C.**

Model 3 – Remission Model

**Appendix D.**

Effects of time and group on gray matter loss

**Appendix E.**

Results of the exploratory whole-brain analysis

Table S4. Results of the exploratory whole-brain analysis comparing patients and controls (groupxtime interaction)

**Appendix F.**

Confounder analysis: Impact of medication, hospitalizations before baseline, symptom-severity and diagnosis (bipolar disorder vs. major depressive disorder), comorbidities, site, number of episodes

Table S5. Psychotropic medication and clinical course at follow-up

**Appendix G.**

Hospitalization effects on gray matter in patients with major depressive disorder compared to HC.

**Appendix A.**

### **Sample characteristics for the hospitalization and the remission model (model 2 and 3)**

| **Table S1. Metric sample characteristics of the patients samples in the remission and hospitalization model** | | | | | | | | | | | | |
| --- | --- | --- | --- | --- | --- | --- | --- | --- | --- | --- | --- | --- |
|  | **Model 3 (Remission at Follow-Up)** | | | | | | **Model 2 (Hospitalization during Follow-Up)** | | | | | |
|  | Remitted patients  *N* = 23, *M* (*SD*) | | Acute patients  *N* = 15, *M* (*SD*) | | Remission  ANOVA | | Patients without hospitalization  *N* = 15, *M* (*SD*) | | Patients with hospitalization  *N* = 13, *M* (*SD*) | | Hospitalization  ANOVA | |
|  | Baseline | Follow-Up | Baseline | Follow-Up | *p*-value | Post hoc | Baseline | Follow-Up | Baseline | Follow-Up | *p*-value | Post hoc |
| Age | 36.87 (10.68) | 45.83 (10.12) | 37.40  (9.75) | 46.33  (9.29) | .877 | - | 32.40  (8.21) | 42.47  (8.68) | 37.62 (11.57) | 47.31 (10.98) | .189 | - |
| Follow-Up Interval (in months) | / | 107.83 (23.20) | / | 107.33 (27.29) | .953 | - | / | 121.00 (15.00) | / | 117.15 (16.96) | .530 | - |
| **Clinical Characteristics** |  |  |  |  |  |  |  |  |  |  |  |  |
| BDI | 21.10 (11.08) | 5.26  (3.29) | 30.62  (9.57) | 19.08  (7.43) | <.001 | Rem < acute | 16.38 (11.86) | 4.92  (4.34) | 29.00  (7.39) | 15.60  (9.81) | .004 | Non-hosp < hosp |
| HAM-D | / | 2.78  (2.45) | / | 11.67  (8.77) | <.001 | Rem < acute | / | 2.40  (3.29) | / | 10.23 (10.32) | .010 | Non-hsop < hosp |
| YMRS | / | 0.61  (0.85) | / | 0.60  (0.97) | .975 | - | / | 0.73  (0.96) | / | 0.46  (0.78) | .423 | - |
| Number of hospitalizations | 2.11  (1.86) | 2.56  (2.00) | 2.00  (1.49) | 3.50  (5.38) | .089 | - | 1.80  (1.61) | 0 (0) | 2.38  (1.85) | 3.54  (4.63) | .006 | Non-hosp < hosp |
| Duration of hospitalizations (in weeks) | 5.75  (7.51) | 1.64  (3.82) | 8.93  (7.35) | 8.45  (11.28) | .068 | - | 4.133  (2.99) | 0 (0) | 10.06  (9.75) | 8.76  (9.81) | .003 | Non-hosp < hosp |
| Medication index | - | 0.83  (1.23) | - | 1.72  (1.99) | .083 | - | - | 0.65  (0.93) | - | 2.40  (2.06) | .004 | Non-hosp > hosp |
| *Note.* Hospitalization data and young mania rating scale are only available for the Münster site. Hamilton depression rating scale and young mania rating scale were unavailable for many participants at baseline and are therefore not reported. *P*-values are reported for ANOVA with subsequent post hoc *t*-tests and between-subjects contrasts of a repeated measures ANOVA if measurements at Baseline and Follow Up were available.  *Abbreviations:* BDI = Beck Depression Inventory I/II; HAM-D = Hamilton Depression Rating Scale, YMRS= Young Mania Rating Scale. | | | | | | | | | | | | |

### **Sample characteristics for each site**

##### **Table S2. Sociodemographic, questionnaire and clinical data of study participants in Münster**

|  | **MDD (N=22)**  **M(SD)** | **BD**  **(N=6)**  **M(SD)** | **HC**  **(N=24)**  **M(SD)** | **P‐value according to χ 2‐tests or t‐tests between clinical groups** | **P‐value according to χ 2‐test or ANOVA between all groups** |
| --- | --- | --- | --- | --- | --- |
| Age at baseline | 35.1  (10.8) | 34  (7.5) | 29.8  (9.7) | .827 | .205 |
| Age at follow-up | 44.8  (10.7) | 44.3  (7.1) | 40.2  (10.5) | .918 | .297 |
| Interscan interval in months | 117.2  (14.8) | 126.5  (18.3) | 125.2  (18.4) | .207 | .233 |
| Sex (male/female) | 14/8 | 1/5 | 8/16 | .099 | .087 |
| Remitted at follow-up (yes/no) | 15/7 | 3/3 | - | .41 | - |
| hospitalized in interval (yes/no) | 9/13 | 4/2 | - | .262 | - |
| BDI at baseline | 18.9  (11.5) | 32.4  (5.7) | 2.7  (2.4) | .021^a^ | <.001^a^ |
| BDI at follow-up | 8.8  (9.3) | 12.5  (8.4) | 3.4  (2.6) | .396 | .005^a^ |
| HDRS at follow-up | 6.1  (8.3) | 6.0  (8.9) | 2.0  (3.7) | .991 | .107 |
| YMRS at follow-up | 0.8  (0.9) | 0.0  (0) | 0.6  (1) | .053 | .205 |
| Antidepressant medication at baseline  (yes/no) | 10/5 | 6/0 | - | .105 | - |
| Antidepressant medication at follow-up  (yes/no) | 8/14 | 4/2 | - | .184 | - |

MDD = patients with Major Depressive Disorder; BD = patients with Bipolar Disorder; HC = healthy controls; SD = standard deviation; BDI= Beck Depression Inventory; HDRS = hamilton depression rating scale; YMRS = young mania rating scale.

^a^Significant at statistical threshold P < 0.05.

##### **Table S3. Sociodemographic, questionnaire and clinical data of study participants in Dublin**

|  | **MDD (N=10)**  **M(SD)** | **HC**  **(N=13)**  **M(SD)** | **P‐value according to χ 2‐tests or t‐tests between groups** |
| --- | --- | --- | --- |
| Age at baseline | 43.4  (7.9) | 40.9  (15.5) | .650 |
| Age at follow-up | 49.7  (8.3) | 47.2  (15.3) | .641 |
| Interscan interval in months | 75.2  (12) | 76  (9.9) | .862 |
| Sex (male/female) | 3/7 | 4/9 | .968 |
| Remitted at follow-up (yes/no) | 5/5 | - | - |
| BDI at baseline | 31.7  (6.6) | 2.1  (2.1) | <.001^a^ |
| BDI at follow-up | 13.3  (7.8) | 0.4  (0.7) | <.001^a^ |
| HDRS at follow-up | 7  (2.6) | 1.3  (2.1) | <.001^a^ |
| Antidepressant medication at baseline  (yes/no) | 9/1 | - | - |
| Antidepressant medication at follow-up  (yes/no) | 1/9 | - | - |

MDD = patients with Major Depressive Disorder; BD = patients with Bipolar Disorder; HC = healthy controls; SD = standard deviation; BDI= Beck Depression Inventory; HDRS = hamilton depression rating scale.

^a^significant at statistical threshold p < .05.

## **Appendix B.**

### **Magnetic resonance imaging parameters**

###### **Münster**

T1-weighted high-resolution anatomical images of the head were acquired (Gyroscan Intera 3T, Philips Medical Systems, the Netherlands) at both time points using a three-dimensional fast gradient echo sequence (turbo field echo), repetition time = 7.4 ms, echo time = 3.4 ms, flip angle = 9°, two signal averages, inversion prepulse every 814.5 ms, acquired over a field of view of 256 mm (feet-head) x 204 mm (anterior-posterior) x 160 mm (right-left), frequency encoding in feet to head direction, phase encoding in anterior-posterior and right-left direction, reconstructed to voxels of 0.5 mm × 0.5 mm × 0.5 mm.

###### **Dublin**

Magnetic resonance images were obtained with a Philips Achieva MRI scanner operating at 3T at baseline and follow-up using a three-dimensional fast gradient sequence (turbo field echo), repetition time = 8.5 ms, echo time = 3.9 ms, flip angle = 8°, 1 signal averages, acquired over a field of view of 256 mm (feet-head) x 256 mm (anterior-posterior) 160 mm (right-left), frequency encoding in feet to head direction, phase encoding in anterior-posterior and right-left direction, reconstructed to voxels of 1 mm × 1 mm × 1 mm.

### **VBM Preprocessing**

To perform a voxel-based morphometry, structural images were preprocessed and segmented using the CAT12 toolbox (version CAT12.7, r1720; http://dbm.neuro.uni-jena.de/cat/) implemented in SPM12. Preprocessing was performed using the longitudinal model to detect larger changes which is indicated for longer time periods, because it includes additional warping and modulation steps. Other parameters remained at default settings, normalization of the segmented images into MNI space were conducted using the Geodesic Shooting algorithm. Finally, the Gray-Matter segments were smoothed with a Gaussian Filter (8 mm full-width half-maximum, FWHM).

For quality control, the gray matter segments were carefully checked using the check homogeneity function and outliers have been visually inspected. In consequence, 12 subjects had to be removed from the sample retrospectively: 3 due to excessive head movement, 9 due to inadequate image quality resulting from other artifacts or strong noise. From the initial sample of 87 subjects, 75 subjects remained in the final sample after quality control.

## **Appendix C.**

### **Model 3 – Remission Model**

The *remission model* (model 3) contained 23 patients that were in full remission at follow up (12 women and 11 men; mean [SD] age, 45.83 [10.12] years) and 15 patients that were acutely depressed or only partially remitted at follow up (8 women and 7 men; mean [SD] age, 46.33 [9.29] years). A total of 37 healthy controls (25 women and 12 men; mean [SD] age, 42.65 [12.63] years) were also included. This analysis included all participants from the Münster and the Dublin site.

Model 3 was calculated because the Dublin patients were all outpatients and we therefore could not use hospitalization as a measure of course of disease in model 2. In model 3, complete remission at follow-up was used as a measure for a benevolent disease course and a partly remitted or acute episode was used as a measure for a severe disease course. We assumed that patients who have a new episode after 9 years or have not yet fully recovered (e.g., are in partial remission) also tend to have a more severe disease course with significant impairment.

The analysis revealed a significant group x time interaction: Patients who were partly remitted or in acute depression at follow-up lost more volume in the hippocampal and insula (ROI analysis) compared to healthy controls (right insula: t (71) = 3.76, p_FWE_ = .034, k =1, x = -21, y = -12, z = -12; left hippocampus: t (71) = 3.92; p_FWE_ = .039, k = 9, x = 27, y = 12, z = -21). Patients in full remission did not differ significantly from healthy participants or acute patients in volume changes from baseline to follow up (all p_FWE_ > .42). The ROI analysis of the DLPFC as well as the whole-brain analysis yielded also no significant results surviving our rigorous and conservative control for alpha inflation (all p_FWE_ > .62).

## **Appendix D.**

### **Effects of time and group on gray matter loss**

The results of model 1 showed a significant effect of time for the whole-brain cluster (F(1,73) = 74.386, p < . 001, with higher brain volume at baseline than at follow up (Baseline - Follow-Up: M = 0.0277, SD = 0.0293, see also Figure 1). This effect was also observable for the hippocampus (F(1,73) = 26.506, p < .001) and the insula ROI-analyses (F(1,73) = 207.054, p < .001). There were no main effects of group in model 1 (whole-brain: F(2,73) = 0.173, p = .679; hippocampus: F(2,73) = 0.207, p =650; insula: F(1,73) = 0.038 , p = .847).

Model 2 revealed significant time effects as well (hippocampus: F(1,49) = 17.281, p < .001, insula: F(1,49) = 39.779, p <.001) with higher volume at baseline than at follow up (e.g., insula: Baseline – Follow Up: M = 0.02566, SD = 0.0367, see also Figure 2). Again, there was no main effect of group (hippocampus: F(2,49) = .290, p = .749, insula: F(2,49) = 1.122, p = .334).

## **Appendix E.**

### **Results of the exploratory whole-brain analysis**

| **Table S4. Results of the exploratory whole-brain analysis comparing patients and controls (groupxtime interaction)** | | | | | | | | |
| --- | --- | --- | --- | --- | --- | --- | --- | --- |
| k | t | p_FWE_ (cluster) | p_FWE_ (peak) | p_unc_ | x | y | z | region |
| 3411 | 5.25 | <.001 | .018 | <.001 | 24 | 9 | -21 | R temporal pole, insula, hippocampus |
| 946 | 4.89 | .042 | .059 | .007 | -22 | -9 | -12 | L hippocampus, amygdala |
| 1076 | 4.85 | .027 | .067 | .004 | 14 | -72 | -27 | R Cerebellum |
| 588 | 4.75 | .154 | .092 | .026 | -42 | -6 | -46 | L IFG |
| 914 | 4.71 | .047 | .102 | .008 | -15 | -75 | -36 | L Cerebellum |
| 347 | 3.87 | .036 | .491 | .078 | -48 | -12 | -2 | L STG, Insula |
| *Note.* The model tested the hypothesis that patients lose more volume than controls in the interscan-interval as a directed groupxtime interaction controlling for age. | | | | | | | | |
| *Abbreviations.* k = clustersize, fwe = family-wise-error corrected, unc = uncorrected, R = right, L = left, STG = superior temporal gyrus, IFG = inferior temporal gyrus | | | | | | | | |

## **Appendix F.**

### **Confounder analysis: Impact of medication, hospitalizations before baseline, symptom-severity, diagnosis (bipolar disorder vs. major depressive disorder) and comorbidities**

Patients with MDD and BD did not differ in their gray matter decline over time (whole brain: t (36) = - 0.603, p = .55; hippocampus t (36) = 1.099, p = .28, insula: t(36) = -1.257, p = .22).

Gray matter changes were also not related to the previous course of disease measured with the number of hospitalizations at baseline in the Münster sample (hippocampus: r_s_ = -.007, p = .49, insula: r_s_ = .187, p = .17, whole-brain: r_s_ = .209, p = .14) and the symptom-severity of the episode at baseline measured with the BDI (hippocampus: r = .124, p = .25, insula: r = -.037, p = .42, whole-brain: r = .195, p = .19) in the patient sample.

Gray matter changes in the hospitalization analysis were also uncorrelated with psychotropic medication intake (hippocampus: r = .318, p = .10; insula: r = .214, p = .14).

Gray matter changes in the hospitalization analysis did not differ between patients with (n = 9) and without life-time comorbidities (n = 19). Comorbidities included anxiety disorders (specific phobia, panic disorder, general anxiety disorder) during the study interval and substance abuse prior to the study interval (cannabis and medication abuse). Since the Levene-test pointed towards heteroscedasticity, we report adjusted degrees of freedom (insula: t (9.908) = -1.890, p = .10; hippocampus:  t (10.894) = -0.878 , p = .39).

Number of depressive episodes were highly correlated with number and duration of hospitalization (number: r_s_= .349, p = .034, duration: r_s_ = .418, p = .013). The number of depressive episodes reported in the interval ranged between 2 to 22 episodes. Also, the number of depressive episodes was strongly correlated with gray matter loss of the insula in the Münster sample (r_s_ = .539, p = .002), but not with hippocampal volume loss (r_s_ = .261, p = .090).

Gray matter changes in our three clusters (whole-brain, hippocampus, insula) across sites were not significantly different across sites (all p’s  > .442).

| **Table S4*.*** *Distribution of psychotropic medication and course of illness at follow up* | | | | | |  | |
| --- | --- | --- | --- | --- | --- | --- | --- |
|  | |  | Remission Model 3 | | Hospitalization Model 2 | | |
| Medication | |  | patients without remission  (n=15) | patients with remission  (n=23) | patients with hospitalization  (n = 13) | | patients without hospitalization  (n=15) |
| Antidepressant, No, % | |  |  |  |  | |  |
|  | SSRI |  | 4, (27) | / | 4, (31) | | / |
|  | SNRI |  | 2, (13) | 5, (22) | 4, (31) | | 3, (20) |
|  | NaSSa |  | / | 1, (4) | 1, (8) | | / |
|  | NDRI |  | / | 1, (4) | 1, (8) | | / |
|  | Tricyclic antidepressant |  | / | 2, (9) | 1, (8) | | 1, (7) |
| Antipsychotics, No, % | |  | 2, (13) | 1, (4) | 3, (23) | | / |
| Mood Stabilizers, No, % | |  | 2, (13) | 6, (26) | 2, (15) | | 3, (20) |
| Other, No, % | |  | 2, (13) | 2, (9) | / | | / |
| Medication Index, M (SD) | |  | 1.72 (1.99) | 0.83 (1.22) | 2.40 (2.06) | | 0.65 (0.93) |
| Depressive episodes*, M (SD) | |  | NR | NR | 6.15 (4.18) | | 3.67 (4.87) |
| Comorbidities, No, % | |  | NR | NR | 5, (38) | | 4, (27) |

*Note.* Psychotropic medication and clinical course data based on self-report. Comorbidities were assessed using the structured clinical interview (SCID-I) based on DSMIV-criteria and included lifetime diagnoses of substance abuse (cannabis, medication; both prior to study participation) and anxiety disorders such as specific phobia, panic disorder and general anxiety disorder.

*Depressive Episodes at follow-up were calculated from the lifetime episodes reported at baseline and follow-up by the respective participant.

*Abbreviations.* SSRI = serotonin-reuptake inhibitor, SNRI = selective serotonin and noradrenalin reuptake inhibitor, NaSSa = Noradrenergic and Specific Serotonergic Antidepressant, NDRI = Noradrenalin-Dopamine Reuptake Inhibitor, NR = not reported in the Dublin sample, therefore not indicated in the remission model

## **Appendix G.**

### **Hospitalization effects on gray matter in patients with MDD compared to HC**

Excluding all BD-patients from the analysis revealed a similar pattern of results for model 2. In particular, results were even more pronounced: MDD-patients with hospitalization lost more volume in the insula and hippocampus (Hippocampus: x = -18, y = -10, z = -12, k = 1, t (43) = 3.84, p_FWE_ = .043, Insula: x = 32, y = 14, z = -21, k = 1, t (43) = 4.00, p_FWE_ = .049) than healthy participants. In addition, MDD-patients with hospitalizations in the interval also lost significantly more volume in the left hippocampus compared to MDD-patients without hospitalizations (t (43) = 4.03, k = 12, p_FWE_ = .026, x = -32, y = -10, z = -20). In model 1, there was no significant groupxtime interaction surviving our rigorous control for alpha inflation, which means that patients with MDD did not differ from healthy controls in their gray matter loss, however, patients with hospitalizations differed from healthy controls and from patients without hospitalizations.
